# Mass Spectrometry-Based Biomarkers to Detect Prostate Cancer: A Multicentric Study Based on Non-Invasive Urine Collection without Prior Digital Rectal Examination

**DOI:** 10.3390/cancers15041166

**Published:** 2023-02-11

**Authors:** Maria Frantzi, Zoran Culig, Isabel Heidegger, Marika Mokou, Agnieszka Latosinska, Marie C. Roesch, Axel S. Merseburger, Manousos Makridakis, Antonia Vlahou, Ana Blanca-Pedregosa, Julia Carrasco-Valiente, Harald Mischak, Enrique Gomez-Gomez

**Affiliations:** 1 Department of Biomarker Research, Mosaiques Diagnostics GmbH, 30659 Hannover, Germany; 2 Experimental Urology Department of Urology, Medical University of Innsbruck, 6020 Innsbruck, Austria; 3 Department of Urology, University Hospital Schleswig-Holstein, Campus Lübeck, 23538 Lübeck, Germany; 4 Systems Biology Center, Biomedical Research Foundation, Academy of Athens, 11527 Athens, Greece; 5 Maimonides Biomedical Research Institute of Córdoba, Department of Urology, University of Cordoba, 14004 Cordoba, Spain; 6 Institute of Cardiovascular and Medical Science, University of Glasgow, Glasgow G12 8TA, UK

**Keywords:** biomarkers, machine learning, omics, prostate cancer, proteomics, urine

## Abstract

**Simple Summary:**

Prostate cancer is the most frequent cancer type and one of the leading causes of death in men globally. Multiple biomarkers analyzed in urine have been proposed for detecting prostate cancer, in an effort to reduce unnecessary and invasive biopsies. Nevertheless, these biomarkers are based on sampling after prior digital rectal examination and/or prostate massage. Considering the need for more convenient urine sampling, in this study, we investigated endogenous urinary peptides in patients with prostate cancer compared to those with non- malignant (non- cancerous) prostatic diseases. A multidimensional biomarker model was developed based on 181 significant peptides that can detect whether a patient has high probability to bear a tumor in the prostate. Based on the results, the biomarker model including 181 biomarkers showed good accuracy in detecting prostate cancer and has the potential to improve clinical management of men with a suspicion of prostate cancer, by reducing the need for invasive biopsies.

**Abstract:**

(1) Background: Prostate cancer (PCa) is the most frequently diagnosed cancer in men. Wide application of prostate specific antigen test has historically led to over-treatment, starting from excessive biopsies. Risk calculators based on molecular and clinical variables can be of value to determine the risk of PCa and as such, reduce unnecessary and invasive biopsies. Urinary molecular studies have been mostly focusing on sampling after initial intervention (digital rectal examination and/or prostate massage). (2) Methods: Building on previous proteomics studies, in this manuscript, we aimed at developing a biomarker model for PCa detection based on urine sampling without prior intervention. Capillary electrophoresis coupled to mass spectrometry was applied to acquire proteomics profiles from 970 patients from two different clinical centers. (3) Results: A case-control comparison was performed in a training set of 413 patients and 181 significant peptides were subsequently combined by a support vector machine algorithm. Independent validation was initially performed in 272 negative for PCa and 138 biopsy-confirmed PCa, resulting in an AUC of 0.81, outperforming current standards, while a second validation phase included 147 PCa patients. (4) Conclusions: This multi-dimensional biomarker model holds promise to improve the current diagnosis of PCa, by guiding invasive biopsies.

## 1. Introduction

Prostate cancer (PCa) is the most frequently diagnosed cancer among men, with approximately 1.5 million newly diagnosed cases worldwide [1], also showing particularly high incidence rates in Europe and Northern America [2]. These high incidence rates are largely attributed to the wide application of prostate-specific antigen (PSA) testing and the aging population [3]. Moreover, although this malignancy is diagnosed in about one out of five men during their lifetime, 78% survive PCa for ten or more years [4], with many men presenting with low-risk indolent disease, which is not likely to rapidly progress to lethal disease.

Diagnosis of PCa is confirmed based on the histopathological verification of tumor presence in prostate biopsies, after a positive result of digital rectal examination (DRE), or after elevated PSA levels and more recently, after a positive or suspicious result of multiparametric magnetic resonance (mpMRI) [3]. Nevertheless, PSA is not accurate enough (as specificity is low), with only less than half (~40%) of all patients with elevated PSA serum levels (≥4 ng/mL) ending up positively confirmed with PCa after biopsy [5]. At the same time, DRE has been reported to be subjective, while suspicious findings that are found during DRE can later disappear [6]. Additionally, mpMRI’s low specificity [7] results in high number of false-positive indications that will have to undergo biopsy, while altogether there is a large degree of inter-reader variation [8], with the interpretation performance found to be highly dependent on the radiologist’s prior experience [9]. Better guidance of invasive biopsies through non-invasive means is thus necessary to reduce over-diagnosis and over-treatment [10,11,12,13], particularly for low-risk indolent PCa.

Several single- or few-biomarker assays based on urinary analysis, such as prostate cancer antigen-3 (PCA3) [14], SelectMDx [15], Mi-Prostate Score [16] and ExoDx [17] have been commercially available for detection of PCa, but have not yet been integrated into clinical guidelines or implemented in healthcare systems [3]. These tests are based on sampling procedures after prior DRE (or prostate massage), building on the principle that the prostate, as a secretory organ, excretes cells, extracellular vesicles, and other molecules in the urethra in the form of prostatic secretions. In an effort to reduce discomfort and increase convenience of the sampling procedure, in this study we propose the transition of biomarker investigations to urine samples that have been collected without prior intervention (i.e., DRE or prostate massage). The clinical goal is to discover and validate urinary biomarkers, which in combination have the potential to improve PCa detection.

Following the above sampling hypothesis, a proof of principle study was previously published focusing, however, on biomarkers to discriminate significant PCa through the application of Capillary Electrophoresis coupled to Mass Spectrometry (CE-MS) [18]. CE-MS has appeared in recent years as a promising hybrid technology that is based on the application of capillary electrophoresis (CE) instead of liquid chromatography allowing for sensitive (up to 1 fmol) and high-resolution low molecular weight protein (up to 20 kilodalton) and peptide separation, before mass spectrometry analysis (MS) [19]. Through the application of CE-MS, high resolution low-molecular weight protein/peptide profiles from >800 patients had been previously investigated for biomarker features related to significant PCa (Gleason score, GS ≥ 7) compared to low grade PCa (GS 6). Based on the previously published data, 19 peptide biomarkers were integrated in a machine learning model that was developed to discriminate significant PCa. The 19-biomarker model was further validated in an independent clinical cohort [20] resulting in an accuracy of 81%.

Following these first published results [18,20], in the present study we expand the investigation of the low-molecular weight proteome (peptidome) to molecularly map differences in the urinary profiles between PCa patients with different disease stages and those with non-PCa etiologies with the aim to better understand the disease background and also propose biomarkers to more accurately guide PCa detection without the need of prior DRE.

## 2. Materials and Methods

### 2.1. Patient Population and Characteristics

This study was performed in line with the REMARK Reporting Recommendations [21] and the recommendations for biomarker identification and reporting for clinical proteomics [22]. Development cohort (Discovery and 1st validation phase): A case-control study was performed on patients with a clinical suspicion of PCa that were scheduled for a transrectal ultrasound (TRUS)-guided biopsy of the prostate during the period from 2013 until 2015 at University Reina Sofia in Cordoba. An ethical approval was granted by the Reina Sofia Hospital Research Ethics Committee (protocol code 30/1992), while informed consent was obtained from all participants of this study. The clinical group consisted of patients recommended for a TRUS-guided biopsy of the prostate, in line with the clinical guidelines and as previously reported [23]. In brief, patients at risk for PCa included: a) those with suspicious findings upon DRE, b) those with PSA serum levels of >10 ng/mL, or c) with PSA 3–10 ng/mL (with concomitant free PSA ratio <25–30%), and d) those patients that underwent previous biopsies and showed a persistent suspicion of PCa. During TRUS-guided prostate biopsy, 12 cores in patients undergoing the first biopsy procedure, and a minimum of 16 biopsy cores for those who had a previous biopsy were obtained. In total, 823 patients were included, for which clinical and biochemical data for all main variables (such as PSA, DRE, number of previous biopsies and treatment) were available. The patient cohort characteristics are summarized in Table 1 and the full clinical and laboratory data were collected and are presented in the Supplementary Table S1. 2nd validation cohort: Additional validation was performed in 147 patients that attended Innsbruck Medical University and were scheduled for a TRUS-guided biopsy of the prostate, following a suspicion for PCa presence based on prior suspicious DRE results, high PSA levels and/or persistent suspicion of PCa after previous biopsies, according to previously described criteria [24]. Patient recruitment took place during the period between 2005 and 2012, when mpMRI had not yet been recommended at routine practice. An ethical approval was granted by the local ethics committee at Innsbruck Medical University (protocol code 11438/2017) and informed consent was obtained from all participants of this study. During TRUS-guided prostate biopsy, 15 biopsy cores were obtained. The biopsy tissue specimens were evaluated by a uro-pathologist according to International Society of Urological Pathology 2005 modified criteria [25]. D’Amico classification utilizing Gleason Score (GS), PSA criteria [3,26] and T-stage were applied to classify the PCa patients into risk groups (low, intermediate and high). The patient cohort characteristics are summarized in Table 1 and the full clinical and laboratory data are presented in Supplementary Table S2. A comparative analysis for the clinical and biochemical variables between the PCa patients and the non-PCa groups, including patients with non- malignant prostatic diseases such as benign prostatic hyperplasia (BPH), those presenting with prostatic intraepithelial neoplasia (PIN) and/or atypical small acinar proliferation (ASAP) is provided in Table 2.

### 2.2. Sample Collection and Processing

Patients donated urine before undergoing a prostate biopsy and it was stored at −80 °C until further processing. There was no DRE or prostate massage performed before urine sampling. Urine preparation and peptide extraction prior to mass spectrometry was performed, according to previously published standard operating protocols for sample preparation and data acquisition, described in detail in [27].

For assessing reproducibility of the CE-MS technique, a standard human urine sample was analyzed as previously described in detail [28]. The standard urine sample was created as a result of pooling multiple midstream morning urine collections donated by eight female healthy volunteers. Morning urine collections were conducted without any requirements relating to specific diet or relevant to menstrual cycle timepoints. Only absence of menstruation was required by the volunteers. The urine collection protocol is in agreement with a “standard protocol for urine collection” developed by the Human Urine and Kidney Proteome Project and European Kidney and Urine Proteomics COST Action (EuroKUP) network [28]. No further additives such as protease or phosphatase inhibitors were included and there was no adjustment of the pH.

The extraction of peptides from the urine samples was conducted by diluting 700 µL urine aliquots with an equal volume (700 µL) of alkaline buffer containing 2 M urea, 10 mM NH_4_OH and 0.02% SDS (pH 10.5), as previously reported [18]. Details of the protocol have been provided before [18], with main steps including first an ultracentrifugation by Centrisart filters (20 kDa MWCO; Sartorius, Göttingen, Germany) to isolate naturally occurring peptides and small proteins <20kilodalton and to eliminate urea, electrolytes, salts and other urine matrix effects. Subsequently, 1.1 mL of the filtrate was applied on PD-10 columns (GE Healthcare, Munich, Germany) after being equilibrated with 0.01% NH_4_OH in high-performance liquid chromatography (HPLC)-grade H_2_O (Roth, Germany). After a rinsing step with 1.9 mL of 0.01% NH_4_OH in H_2_O, 2 mL of HPLC-grade H_2_O was applied, and the resulting eluate was lyophilized and stored until further processing as previously described [29].

### 2.3. Mass Spectrometry Analysis and Post-Acquisition Data Processing

CE-MS analysis and data processing was performed according to ISO13485 [30]. Peptide separation was performed through a P/ACE MDQ capillary electrophoresis system (Beckman Coulter, Fullerton, CA, USA) coupled with a Micro-TOF MS (Bruker Daltonic, Bremen, Germany). In detail, the resuspended peptide extracts (250 nL) were injected hydrodynamically at 2.0 psi for 99 s. Separation of the peptides through the silica capillary was performed under application of reverse polarity at 25 kV for the first 30 min, and with increasing pressure (up to 0.5 psi) for another 34 min [29]. Details regarding the protocol for CE analysis and the composition of the acetonitrile-based running buffer were reported previously [29]. Regarding the coupling interface, an electrospray ionization interface from Agilent Technologies (Palo Alto, CA, USA) was set to a potential of −4.0 to −4.5 kV. Spectra were collected every three seconds and the recordings included an m/z range between 350 and 3000 [29]. Deconvolution of mass spectrometry ion peaks presenting at different charge states was performed through the proprietary software MosaiquesVisu [30,31]. A mass spectrometry peak list for each sample was defined by the molecular mass (kDa), normalized migration time (min) and normalized signal intensity (AU) per identified peptide [31]. Normalization of the relative peptide intensity was performed on the basis of twenty nine collagen fragments that are stably detected in urine independently of the disease or health status [32]. The list of the twenty nine collagen fragments that serve as internal standards is provided in Supplementary Table S3. The peptide lists along with their normalized intensities were saved at an internal database developed based on Microsoft SQL principles [19]. Transformation of the data (log-transformation) was performed prior to the statistical analysis as previously described [33].

### 2.4. Peptide Sequence Assignment

Sequencing of the endogenous peptide fragments is based on matching of the ion peaks obtained with the peptide sequences obtained by liquid chromatography-mass spectrometry analysis (LC-MS/MS) on the basis of the correlation of mass between the two instruments. Further validation of the obtained peptide identifications was based on the assessment of the peptide charge and the CE-migration time results, as reported in detail in [30]. The amino acid sequences were obtained by performing MS/MS analysis using either a PACE CE or a Dionex Ultimate 3000 RSLS nanoflow system (Dionex, Camberley, UK) coupled to an Orbitrap Velos instrument (Thermo Fisher Scientific Inc., Boston, MA, USA), as previously described [34]. The mass spectrometer was operated in MS/MS mode scanning from 350 to 1500 amu. The fragmentation method was HCD at 40% collision energy. Details on the selection of the multiply charged ions for CE and LC-MS/MS as well as the detection limit thresholds have been provided previously [35]. Sequencing was based on database search against Uniprot human non-redundant database (fasta file version from 20 June 2019) using Proteome Discoverer 2.4 (activation type: HCD; precursor mass tolerance: 5 ppm; fragment mass tolerance: 0.05 Da) without enzyme specificity. No fixed modification was selected. Oxidation of proline and methionine (indicated with ‘p’ and ‘m’) as well as deamidation (indicated with ‘q’) were set as variable modifications. Confidence levels based on Xcorr and ranking are detailed in [36].

### 2.5. Statistical Analysis

Statistical analysis by performing a case-control comparison was conducted in the discovery group of 413 patients. This included 139 men with confirmed PCa and a control group including 274 patients with non-PCa etiologies. This approach has been previously reported in other biomarker studies [37]. 410 patients were further grouped in the 1st validation group. Discovery and validation grouping was based on a random split to ensure that each group is properly represented in all patient groups. An additional 2^nd^ validation was performed in 147 PCa patients. Potential interfering clinical variables and clinical bias was assessed within discovery and validation sets and also between the PCa case and non-PCa groups, by Mann–Whitney non-parametric test and Chi-squared test for numerical and categorical variables, respectively (Table 1 and Table 2). The CE-MS urine profiles were compared for differences in peptide abundance between PCa and non-PCa groups in the discovery set by applying the Wilcoxon rank sum test [33]. A frequency threshold of 90% in at least one of the two groups was applied. Statistical correction of the estimated *p* values for multivariate testing was performed based on the Benjamini–Hochberg method [38]. In parallel, correlation analysis using Spearman rank correlation test was performed in the development cohort of 823 patients with PCa, and non-PCa patients. The analysis is built on the hypothesis that cancer progresses as a continuum, and the features that are truly associated with this process are gradually and consistently changed. Therefore, Spearman rank correlation test was applied to define molecular features of which abundance is significantly associated with progression, as represented by increased GS. A *p*-value <0.05 was considered statistically significant. Visual depiction of the compiled urinary polypeptide spectra for PCa patients and the corresponding non-PCa group was performed for each peptide by plotting this based on the normalized migration time (10–55 min) against the molecular mass (0.8–15 kDa). The signal intensity is represented by the height of the peak and corresponds to the mean values of each peptide within the given clinical group.

### 2.6. Machine Learning Model Construction and Optimization

A machine learning model based on the significant peptide biomarkers as derived based on the statistical analysis in the discovery set, was developed by MosaCluster propriatery software (version 1.7.0), which is based on support vector machine (SVM) principles. The biomarker model was optimized in the discovery set, by projecting each biomarker in multidimensional parameter space [31]. In the 1st validation set, the sensitivity and specificity estimates for the SVM-based biomarker model, were calculated based on the number of patients correctly classified as PCa or non-PCa. The optimal cut-off was estimated based on the Youden index statistical test. During the 2^nd^ validation phase, sensitivity estimates were calculated, as the cohort included only PCa patients. The receiver operating characteristic (ROC) plots and the respective confidence intervals (95% CI) were based on exact binomial calculations and were calculated in MedCalc 12.7.5.0 (Mariakerke, Belgium). AUC values were then compared using DeLong tests. Statistical comparisons of the classification scores in the validation cohorts were performed by the Kruskal–Wallis rank sum test using MedCalc. For the assessment of the net benefit for the application of the biomarker model, a decision curve analysis (DCA) was performed, as proposed by Vickers and Elkin [39]. The net benefit was calculated based on the decision threshold at which a person would consider undergoing biopsy. For the AUC and DCA analyses MedCalc 12.7.5.0 (Mariakerke, Belgium) and R version 3.2.3 were used, respectively.

**Figure 1:**
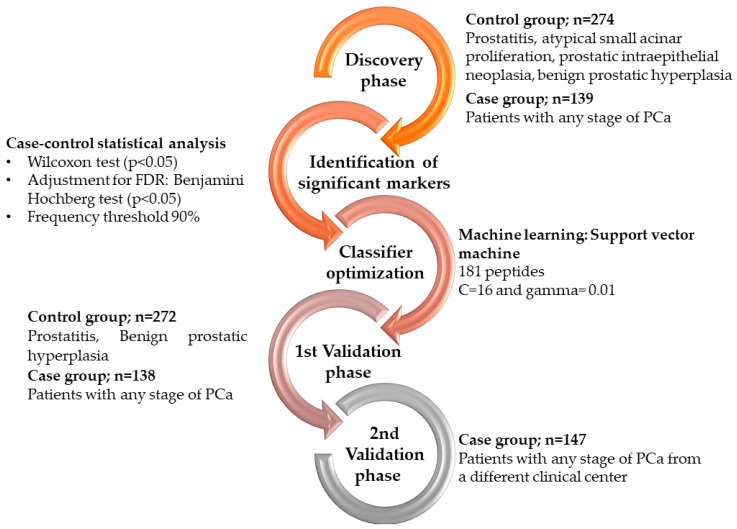
Graphic depiction of the study design and phases for the development and validation of the urinary biomarker model based on CE-MS.

As comparator models, the European Randomised Study of Screening for Prostate Cancer (ERSPC) risk calculator was applied to calculate the risk for PCa detection via: (http://www.prostatecancer-riskcalculator.com/seven-prostate-cancer-riskcalculators, accessed on 1 August 2022), as previously described [23]. The study design, including the different study phases is depicted in Figure 1.

### 2.7. In Silico Protease Prediction and Bioinformatics Analysis

Investigation of protease activity was performed in silico, through the Proteasix (www.proteasix.org, accessed on 1 September 2022) online tool on the basis of proteolytic events that are involved in the generation of the endogenous peptides in urine [40]. In particular, Proteasix analysis was applied to predict the protease activity for the peptides for which sequence information is available and that were identified as significantly associated with PCa progression. The observed proteases that are leading to the cleavage of N- or C-terminus of a peptide were retrieved from CutDB database available at www.cutdb.burnham.org (accessed on 10 September 2022) [41]. Protease/cleavage site associations were retrieved based on matching against the cleavage sites as reported in the literature as well as the probability of a protease cleavage event, based on MEROPS specificity matrices. Activation status of the proteases was calculated as described previously [31]. Gene Ontology terms (biological process, molecular function, cellular compartment), Molecular Signatures Database (MSigDB) hallmark gene set collection associations, and subcellular localization, were retrieved using Metascape [42]. Protein Class was allocated based on the Panther Classification System (http://www.pantherdb.org/ (accessed on 30 September 2022) [43]), while information on protein function was extracted by Uniprot Database. Additional evidence supporting the protein presence was retrieved from NextProt Database [44]. Subsequently, functional enrichment analysis was conducted using Metascape [42], following the default settings. Briefly, terms with *p <* 0.01 (Hypergeometric test), a minimum count of three and an enrichment factor > 1.5 (calculated as the ratio between the observed and the randomly expected counts) were grouped. The protein interaction network was created using STRING v. 9.1 (http://string-db.org/, accessed on 10 October 2022) [45].

## 3. Results

### 3.1. Discovery of Peptides with Significantly Altered Abundance in Urine for PCa

**Figure 2:**
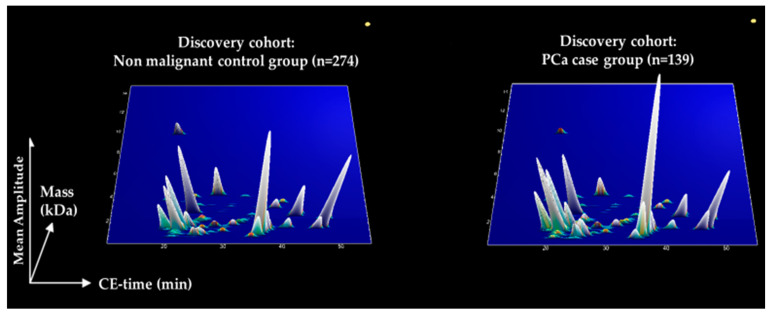
Compiled urinary profiling signatures of PCa patients (n = 139) compared to those with non-PCa etiologies (n = 274). In this plot, each peptide is plotted based on the normalized migration time (10–55 min) on the *x*-axis against the log molecular mass (0.8–15 kDa) on the *y*-axis. The signal intensity is represented by the peak height, corresponding to the mean values of each peptide.

For detecting biomarker peptides specific to PCa, a case-control comparison was performed considering the CE-MS datasets in the discovery set of 413 patients, as visually depicted in Figure 1. The comparison enabled the identification of 181 peptides that demonstrated statistically significant differences (*p* < 0.05, Benjamini Hochberg test; 90% frequency threshold), in their distribution between patients with PCa compared to non-PCa groups (Supplementary Table S3). The schematic representation of the biomarker signature, based on the compiled urinary datasets is comparatively presented in Figure 2. Molecular mass (0.1–12 kDa) is presented on a logarithmic scale and is plotted against normalized migration time (10–55 min), while the peak height depicts the peak intensities based on the average normalized peptide abundance in the compiled patient datasets from the discovery sets. Among the 181 peptide biomarkers, sequences could be matched for 80 peptides, corresponding to 33 unique parental proteins. Most peptide sequences derived from collagen parental proteins. Peptide fragments originating from alpha-1 collagen of types I, II, III, V, VII, XXV, XVI, XXIV, XI, XVII, XXIII, alpha-2 types I, IV, XI, IX and alpha-3 type IV, were most common, while fragments of collagen type (VIII) chain were also detected. Almost all the collagen peptide fragments are of increased abundance by a factor of 1.2 and above in the PCa cases, apart from collagen alpha-1(I) and collagen alpha-1(II). Interestingly, similar to a previous CE-MS study within the collagen peptide sequences, a repetitive motif (pGP) was very frequent [18]. Other peptide sequences were proteolytic products of protein phosphatase 1 regulatory subunit 3A, fractalkine or chemokine (C-X3-C motif) ligand 1, protein S100-A9, uromodulin, albumin, fibrinogen alpha, alpha-1-acid glycoprotein 1, mucin-2, xylosyltransferase 1, polymeric immunoglobulin receptor, matrix Gla protein, beta-2-syntrophin. All peptide fragments from the above proteins were found at higher abundance in urine from PCa patients compared non-PCa group. In contrast, peptides at decreased abundance in urine from PCa patients compared to the non-PCa group, originated from gelsolin, prostaglandin-h2 D-isomerase and insulin-like growth factor II.

### 3.2. Development and Validation of a Biomarker Model Based on CE-MS Significant Peptides for PCa

**Figure 3:**
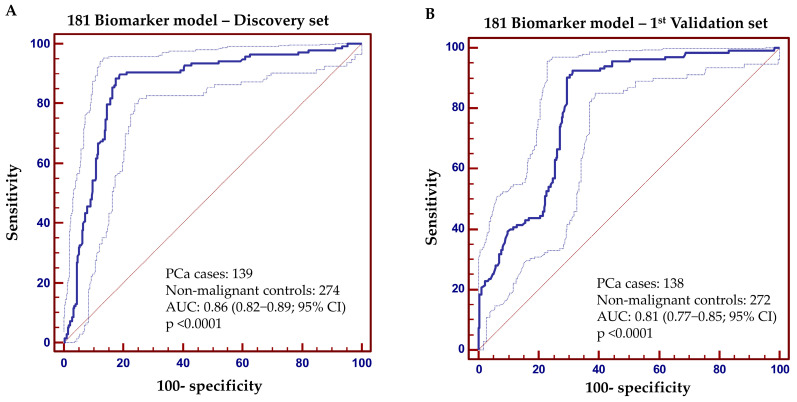
Receiver operating characteristics (ROC) analysis was performed in the (**A**) discovery and (**B**) 1st validation sets, displaying the performance of the 181 Biomarker model for discriminating the group of PCa patients from the non-PCa group. ROC characteristics, such as area under the curve (AUC), 95% confidence intervals (CI), and *p* value are provided for the classification of PCa patients and non-PCa group.

Using the 181 peptides that were identified with statistically significant differences at their abundance in urine between PCa and non-PCa groups, a machine learning algorithm based on SVM, was adopted and optimized to develop a multidimensional biomarker model. After optimization of the SVM-based biomarker model, the optimal parameters were SVM-C: 16 and SVM-gamma: 0.01. Based on these parameters, the estimated AUC value was 0.86 after cross-validation analysis within the discovery set of 413 patients (Figure 3A). Subsequent first validation of the 181-biomarker model in the 1st validation set (n = 410), as proposed in the recommendations for biomarker identification and reporting in clinical proteomics [33], resulted in an overall AUC value of 0.81 (range from 0.77 to 0.85; 95% CI; *p* < 0.0001). Figure 3B presents the ROC curve, which at the pre-defined cut-off of 0.007 resulted in sensitivity levels of 93% (87–96; 95% CI;) and specificity of 69% (63–74; 95% CI), respectively. Considering a prevalence rate of 40% for PCa in this particular clinical cohort, negative predictive value (NPV) was 93.7% while positive predictive value (PPV) was 63.7%. Additional statistical analysis was performed, by application of a post-hoc rank sum test to compare the scores between the PCa case and non-PCa control groups. As depicted in Supplementary Figure S1, the classification of each group differs at the significance level of *p* < 0.000001. Average rank levels were 163.48 and 289.27 for the non-PCa controls and PCa cases, respectively (Supplementary Figure S1).

**Figure 4:**
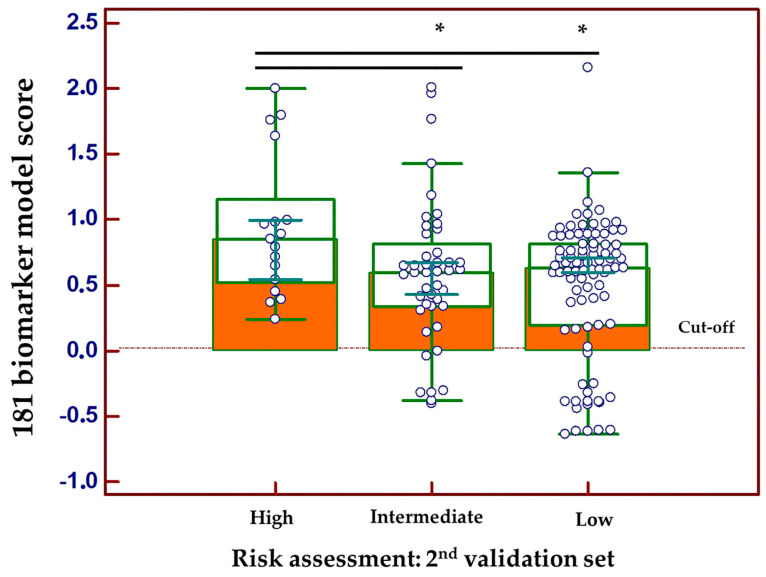
Classification scores, presented in Box-and-Whisker plots and grouped according to the risk group in the second independent validation set consisting of 147 PCa cases. A post-hoc rank-test was performed using the Kruskal–Wallis test. * means; *p* < 0.05.

A second independent validation was performed in 147 patients with PCa recruited at the second clinical center (Innsbruck cohort). The 181 Biomarker model correctly classified 123 of 147 cases, whereas 24 cases were missed. Among the missed PCa cases, there were none from a high-risk group, 7 had intermediate-risk PCa and the remaining 17 had a low-risk tumor. Along these lines, the SVM-based classification score of 181 Biomarker panel was significantly higher in patients with high risk PCa compared to low and intermediate risk (Figure 4; *p* < 0.05; Kruskal–Wallis H-test).

**Figure 5:**
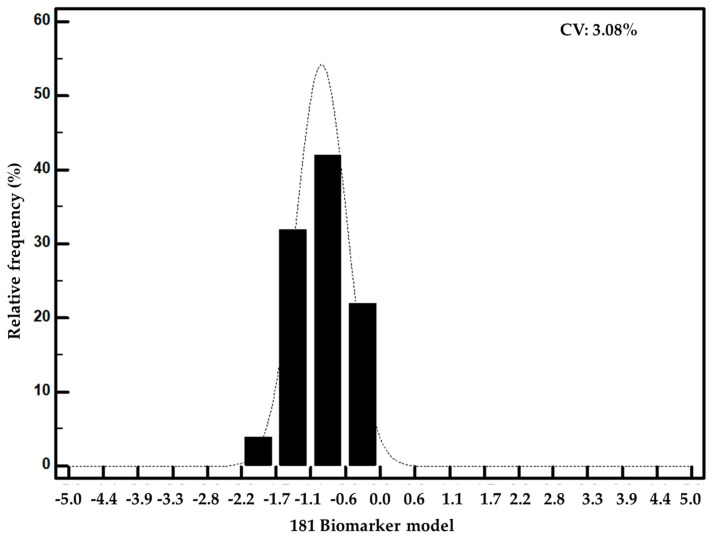
Distribution of classification scores of the 181 Biomarker model for 50 measurements of the standard human urine sample.

Analytical validation of the 181 Biomarker model was additionally performed by investigating the reproducibility of the classification scores in 50 different CE-MS datasets of the standard urine sample over the course of several days. Negative classification scores in the range between −2 and −0.2 were reported distributed normally, as shown in Figure 5. Very low variation was additionally observed (coefficient of variation was estimated at 3%). The classification scores along with the details on the number of identified peptides and the mean intensity are described in Supplementary Table S4.

### 3.3. Comparator Models and Added Value over Clinical Standards

**Figure 6:**
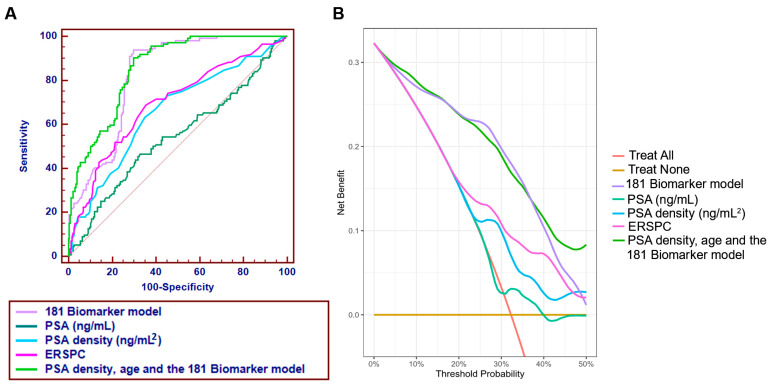
(**A**) Comparative ROC analysis for the main comparator models including the 181 Biomarker model (based on CE-MS), PSA (ng/mL), PSAD (ng/mL^2^), ERSPC and an integrative diagnostic nomogram. (**B**) Decision curve analysis including the above comparator models, to assess the benefit of undergoing a biopsy at the different thresholds.

A direct comparison of the 181 Biomarker model with clinical standards, such as PSA, PSA density and the score based on the ERSPC calculator, was performed in the validation set, as per availability of the underlaying data (i.e., prostate volume). Of note, out of 410 patients, eight patients who had received previous treatment with 5-alpha-reductase inhibitors were excluded from the analysis as the medication may affect PSA levels. Moreover, as information on prostate volume was not available for all PCa patients, the comparison was possible in the validation set including 347 patients. As depicted in Figure 6A, the 181 Biomarker model significantly outperformed the PSA, PSA density and ERSPC, with AUC values at 0.82, 0.54, 0.65 and 0.65, respectively (*p* < 0.0001). Added value of the combination of the comparator models into an integrative diagnostic nomogram, including the 181 Biomarker model was additionally investigated. Logistic regression analysis was performed, including clinical, demographical and omics (CE-MS peptide markers) parameters such as age, 181 Biomarker model, PSA density and ERSCP. Based on the statistical comparison, a significant contribution to the outcome is revealed for age (*p* = 0.006), PSA density (*p* = 0.01), ERSPC (*p* = 0.04) and the 181 Biomarker model (*p* = 0.0001). Combination of these significant variables resulted in an improved AUC value of 0.86, statistically significant (*p* = 0.0027) compared to the 181 Biomarker model. Interestingly, also combination of just the PSA density, age and the CE-based biomarkers (in the form of the 181 Biomarker model score) resulted also in a statistically significant superior performance (AUC of 0.85) than that of the 181 Biomarker model alone (*p* = 0.0058). The latter integrative diagnostic nomogram, because of its simpler construction is more likely to be practical for calculations. The performance characteristics for different threshold points are summarized in Table 3.

For accessing the clinical utility of the 181 Biomarker model, we have additionally performed a decision curve analysis (DCA), as shown in Figure 6B. The DCA analysis showed a high net benefit of the 181 Biomarker model particularly in the lower range of risk thresholds (<50%), compared to clinical variables such as PSA, PSAD and ERSPC. In this case, application of DCA is used to determine whether the 181 Biomarker model can be used as a predictor to make clinical decisions such as performing a biopsy.

### 3.4. Correlation of the Peptide Profiling Data with PCa Progression

To investigate the association of urinary peptides with PCa progression, correlation analysis was performed for the full cohort of 823 patients from the development phase. The statistical analysis revealed 270 sequenced peptides that were significantly correlated with the PCa Gleason score (Supplementary Table S5). When further considering a frequency threshold of at least 30%, 91 peptides were shortlisted. When limiting to parental proteins represented by consistently regulated peptides (≥75%) (Table 4), fragments derived from collagen alpha-1(V) chain and collagen alpha-1(XXII) chain were shown to be positively correlated with disease progression, while negative correlation was observed for collagen alpha-1(I) and collagen alpha-1 (II) chain fragments, confirming the observations from the case- control statistical comparison. Further peptides that were included in the 181 Biomarker model and were also associated with disease progression originated from C-X-C motif chemokine 16 protein, protein S100-A9, matrix Gla Protein, fibrinogen alpha and fractalkine, confirmed with same trend of expression (i.e., elevated in PCa, or as disease progresses). Similarly, gelsolin, collagen alpha-1(III), collagen alpha-1(II) chain were inversely correlated with disease progression and were also included in the 181 Biomarker model as they were found with decreased abundance in urine from patients with PCa compared to the non-PCa group.

### 3.5. Link to Pathophysiology and Dysregulation of Proteases

A link to PCa pathophysiology was attempted by investigating the altered activity of proteases that were involved in the generation of the naturally occurring urinary peptide biomarkers as defined based on the correlation analysis in the full development cohort of 823 patients. For this purpose, the Proteasix online tool [40] was applied to predict the protease activity. Protease/cleavage site associations were retrieved based on matching against cleavage site associations from the literature as well as probability of cleavage by a protease based on MEROPS specificity matrices; the activation status of the proteases was calculated as described previously [31] and also reported in detail in Supplementary Table S6. The analysis resulted in prediction of 41 proteases based on at least 5 cleavage sites. The 10 top-ranked proteases (based on the calculated Xcorr score) exhibiting an increased or decreased activity are reported in Table 5. Positive association with disease progression can be observed for matrix metalloproteinase-20, calpain-2 catalytic subunit, and calpain-2 catalytic subunit, while for cathepsin G and chymase a trend towards negative association was predicted based on the in silico analysis.

**Figure 7:**
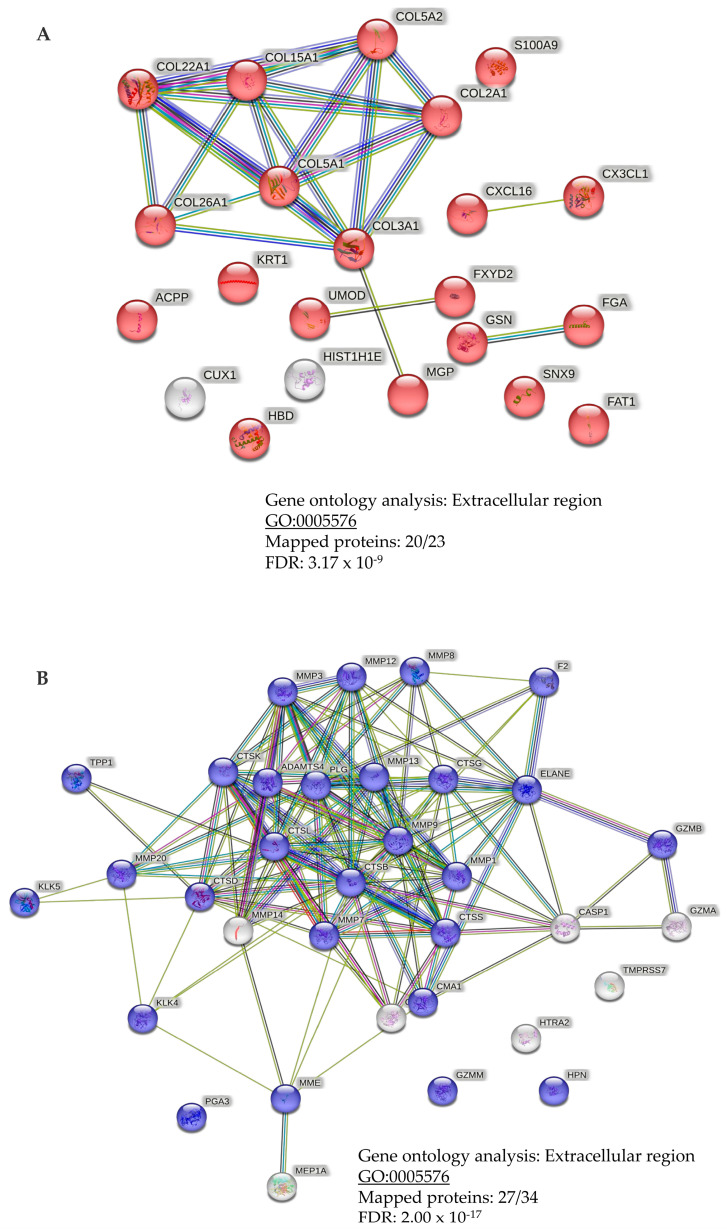
Gene ontology analysis following protein-protein network construction based on STRING underlaying interactome data. Analysis based on (**A**) urinary profiling input list and (**B**) the list of significantly predicted proteases derived from Proteasix analysis.

Subsequent bioinformatics analysis was performed to investigate protein-protein interactions and gene ontology processes based on both the input from the urinary profiling data, as well as the list of predicted proteases. Both gene ontology analyses based on the urinary profiling data and the list of significantly predicted proteases revealed extracellular matrix involvement after mapping of 20 out of 24 proteins based on the urinary profiling data (*p* = 3.17 × 10^−9^; Figure 7A) and mapping of 27 out of 34 proteins based on the Proteasix data (*p* = 2.00 × 10^−17^; Figure 7B), respectively.

## 4. Discussion

Guiding invasive biopsies for detection of PCa through non-invasive means is an unmet clinical need. Several studies focusing on urinary biomarkers have reported promising biomarkers but are all based on urine collection after DRE or prostate massage [14,16]. Building upon our previous study [18], which aimed at the discrimination of significant PCa, in this manuscript we focus on the exploitation of CE-MS profiling datasets towards the development and independent validation of urinary biomarkers that can accurately detect any type of PCa and also to investigate their potential role in PCa molecular pathogenesis. Hence, in this study, a machine learning biomarker model based on 181 peptides was established and validated in independent (validation) cohorts from two different clinical centers. The 181 Biomarker model exhibited a good performance (AUC of 0.81), significantly superior to that of the current clinical standards (PSA, PSA density and ERSPC) [23], while integration of all models in a multiparametric diagnostic nomogram significantly improved the performance (AUCs of 0.85–0.86). An integrative diagnostic nomogram including the 181 Biomarker model, along with age and PSA density has great potential; given the good accuracy (AUC of 0.85), it might be more practical than obtaining ERSPC estimates. These results confirm earlier evidence that biomarker models based on a higher number of biomarkers frequently result in increased stability and performance [33]. Similarly, the fact that an integrative diagnostic nomogram based on different clinical, demographic and omics traits led to significantly improved performance is in line with our previous observations that high complementarity does occur between the different molecular and biochemical biomarkers [20]. Analytical validation is an important aspect when aiming at clinical applications. Regarding CE-MS, biomarker models that are developed based on CE-MS derived peptides, are already used for diagnosis, prognosis and monitoring of complex diseases, as well as for patient stratification in clinical trials [46,47,48]. CE-MS analytical performance has been recently assessed with regards to its inter- and intra-patient reproducibility, variability and efficiency in peptide detection [49]. Reproducibility, repeatability and stability experiments were performed per run and per peptide, indicating CV estimates of >2%. In the present study, reproducibility experiments for the 181 Biomarker model were also performed using 50 repeated analyses of a well-established standard human urine sample according to the standardized operating protocol (SOP) and quality control steps for CE-MS analysis [28]. As expected, none of the measurements scored positive, as the urine sample was derived from a healthy individual, not bearing prostate cancer. In addition, very low variation was observed (CV was estimated at 3%). Compared to other commercially available biomarker tests, the CE-MS based biomarkers resulted in similar or better performance (published AUC estimates for other biomarker tests range between 0.70–0.80) [15,50]. However, the big advantage of this approach is that a prior intervention (DRE or prostate massage) is not required prior to urine sampling. Such a non-invasive diagnostic nomogram can have a potential as a stratification tool in the pathway prior to mpMRI and subsequent biopsy procedure. Unfortunately, paired comparison with the above biomarker tests was not possible, as well as side-by side comparison with mpMRI, as at the time of patient recruitment and urine collection, mpMRI was not yet applied in routine clinical practice. Nevertheless, comparison with mpMRI literature data indicates that the AUC of 0.81 is comparable with the AUC of mpMRI and thus justifies further validation for the biomarkers in a prospectively collected cohort, where paired proteomics and mpMRI data can be acquired and evaluated in comparison and/or in combination. In fact, such a study is currently being organized including Cordoba and Seville University Hospitals (PI22/01769).

In terms of the biomarkers’ sequence and origin, fragments originating from different collagen parental proteins were detected, the majority with increased abundance in the urine from the patients with PCa compared to the non-PCa group. Only collagen alpha-1 (I) and II chains were identified with decreased excretion levels. This result confirms previous studies using the CE-MS platform. Compared to our previously studied biomarker model for distinguishing significant PCa, nine of the 19 peptide biomarkers were significant in this study, following also the same regulation trend [18,20]. Among them were the fragments from fractalkine, protein phosphatase 1 regulatory subunit 3A, collagen alpha-1(I) chain, collagen alpha-1(XVI) chain, collagen alpha-1(XVII) chain, collagen alpha-1(XI) chain, collagen alpha-2(I) chain. The above collagen fragments were also rich in pGP motif, as previously reported [18,20], indicating once more the existence of neutrophil activation in inflamed tissue, as a result of a chemokine binding to C-X-C motif binding signaling [51]. The high abundance of specific collagen peptides is likely reflecting processes of the extracellular matrix components, as a result of tumor invasion and progression. These processes lead to proteolytic products, which are subsequently excreted in urine. This was also supported by the gene ontology analysis upon the construction of the protein-protein interaction networks.

Interestingly, correlation analysis revealed multiple peptide fragments also correlating with disease progression. Fibrinogen peptides (here positively correlating with PCa progression) have been previously reported at high abundance in urine from patients with high-grade PCa, in an integrative urinary investigation including peptidomics and transcriptomics data [52]. Fibrinogen has been previously correlated with progression of urological cancers, as an indicator of tumor related inflammation and angiogenesis [49]. Additionally, prostatic acid phosphatase here reported as inversely correlated with PCa progression, is a phosphatase highly active in seminal plasma but also a known tumor suppressor of PCa through dephosphorylation of ERB2 and deactivation of MAPK-mediated signaling [53]. At the same time, the validity of the in silico prediction is also supported by the existing literature. Many of the predicted proteases have been previously linked to PCa including among others kallikrein-5 [54,55], 72 kDa type IV collagenase [56], calpain-2 catalytic subunit [57], caspase-1 [58], granzyme A [59], chymase [60].

Besides the solid biomarker data, this study is associated with certain limitations. In the involved clinical cohorts, prostate pathology was determined by TRUS-guided biopsy, which is expected to be associated with under- or over-grading compared to the results at radical prostatectomy and is particularly liable to miss small tumors [61]. A second limitation, as also addressed above, is that no paired mpMRI data was available for the PCa groups included in this study as urine samples were collected before mpMPRU was introduced to clinical practice. Yet, based on the literature evidence, while mpMRI can detect over 95% of significant disease it does have a high false positive rate of ~50% [62]. With the reported sensitivity estimates of the 181 Biomarker test, we foresee that combination with mpMRI can reduce the number of biopsies engaged. These results are, therefore, a good starting point for validation of the predicted clinical benefits of the CE-MS based model in a prospective study, including paired mpMRI data (PI22/01769).

## 5. Conclusions

The combination of multiple peptides into a biomarker model based on machine learning improves our ability to detect PCa using urine samples without the need for prior digital rectal examination. The 181 Biomarker model demonstrated good accuracy in detecting PCa and offers a potential for reducing invasive procedures in men that are being scheduled for a biopsy.

## Figures and Tables

**Table 1:** Summary characteristics for the main clinical and biochemical variables for the development cohort (Cordoba Cohort) grouped into a discovery and a validation group, along with the 2nd validation cohort (Innsbruck cohort).

Baseline Characteristics	Discovery Phase (*n* = 413)	1st Validation Phase (*n* = 410)	*p*-Value (1st Validation vs. Discovery)	2nd Validation Phase (*n* = 147)	*p*-Value (2nd Validation vs. Discovery)
Median age (95% CI; yr)	64.0 (63.4–64.8)	64.0 (63.0–65.0)	0.6488 ^¥^	66.0 (64.2–67.0)	0.3414 ^¥^
PSA median (95% CI; ng/mL)	5.4 (5.1–5.8)	5.1 (4.8–5.4)	0.6537 ^¥^	5.2 (4.5–5.9)	0.6298 ^¥^
Digital Rectal Examination (normal/suspicious/NA)	339/74	340/70	0.7676 *	90/20/37	0.3534 *
Previous biopsies (Y/N)	109/304	99/311	0.5258 *		0.8033 *
Prostate volume (95% CI; mL)	36.0 (34–39; *n* = 364)	35.0 (34–37; *n* = 357)	0.4416 ^¥^	40.0 (35–45; *n* = 135)	0.0305 ^¥^
PSA density (95% CI; ng/mL^2^)	0.14 (0.13–0.15; *n* = 364)	0.14 (0.13–0.15; *n* = 357)	0.9379 ^¥^	0.14 (0.12–0.15; *n* = 135)	0.5568 ^¥^
Median urinary creatinine (95% CI; mmol/L)	8.0 (7.3–8.3)	7.8 (7.3–8.3)	0.3696 ^¥^	8.8 (7.6–10.3)	0.1163 ^¥^
Disease pathology					
▪ GS 6	65 (46.8%)	66 (47.8%)	0.9132 *	99 (67.4%)	0.1838 *
▪ GS 3 + 4/ GS 4 + 3	49 (35.3%)/ 14 (10.0%)	46 (33.3%)/ 15 (10.9%)		31 (21.1%) 4 (2.7%)	
▪ GS 8	6 (4.3%)	8 (5.9%)		8 (2.7%)	
▪ GS ≥ 9	5 (3.6%)	3 (2.2%)		5 (5.4%)	
Non-PCa aetiologies					
▪ Benign prostatic hyperplasia; BPH	241 (88.0%)	241 (88.6%)	0.1515 *	-	
▪ Prostatic intraepithelial neoplasia; PIN	18 (6.6%)	16 (5.9%)		-	
▪ Atypical small acinar proliferation; ASAP	15 (5.4%)	15 (5.5%)		-	

^¥^ Mann–Whitney test; * Chi-squared test; Abbreviations: CI—Confidence Interval, GS—Gleason Score; N—No; Y—Yes; yr—Years; PSA—Prostatic Specific Antigen.

**Table 2:** Summary characteristics for the main clinical and biochemical variables for the PCa patients in comparison with the non-PCa group, including patients with non-malignant prostatic diseases.

Baseline Characteristics	Group 1: Non-PCa	Group 2: PCa	*p*-Value Group 1 vs. Group 2
▪ Median age (IQR; yr)	63.0 (57–69)	66.0 (61–71)	<0.0001 ^¥^
▪ PSA median (IQR; ng/mL)	5.1 (3.8–6.9)	5.7 (4.0–8.0)	0.0023 ^¥^
▪ Digital Rectal Examination (Pos/Neg)	60/486	84/193	0.0249 *
▪ Previous biopsies (Y/N)	159/387	49/228	0.8646 *
▪ Prostate volume (IQR; mL)	38.0 (29–52; *n* = 476)	30.0 (22.9–43.1; *n* = 245)	<0.0001 ^¥^
▪ PSA density (IQR; ng/mL^2^)	0.13 (0.09–0.18; *n* = 476)	0.18 (0.13–0.26; *n* = 245)	<0.0001 ^¥^
▪ Median urinary creatinine (IQR; mmol/L)	7.7 (5.5–10.3)	8.0 (5.8–10.6)	0.3636 ^¥^

^¥^ Mann–Whitney test; * Chi-squared test; Abbreviations: IQR—Interquartile Range; N—No; Neg—Negative; PCa—Prostate Cancer; Pos—Positive; Y—Yes; yr—Years.

**Table 3:** Performance characteristics for the proteomics and the clinical comparators at different thresholds.

	181 Biomarker Model		PSAD		ERSPC		Diagnostic Nomogram	
Sensitivity Thresholds	Specificity	95% CI	Specificity	95% CI	Specificity	95% CI	Specificity	95% CI
80.0	71.4	65.1–76.6	37.1	23.4–56.0	40.7	28.2–57.8	72.8	65.4–78.2
90.0	70.4	58.4–77.0	19.2	8.33–34.7	23.0	11.9–37.2	67.5	56.4–74.9
95.0	55.5	32.4–74.1	7.9	3.6–15.6	12.9	2.8–22.5	56.0	40.5–66.7
97.5	31.5	1.2–54.6	4.9	1.6–10.1	4.1	0.7–15.9	45.3	14.0–58.6

**Table 4:** Shortlisted urinary peptides significantly associated with PCa progression. Spearman rank correlation analysis was applied to define molecular features of which abundance is significantly associated with progression, as represented by increased GS.

Mass [Da]	CE-Time [min]	Peptide Sequence	Protein Name	*p*-Value	Spearman’s Rho
1353.66	25.88	PVGpSGKDGANGIpG	Collagen alpha-1(II)	0.0051	−0.098
3718.72	32.42	SGPPGRAGEPGLQGPAGPpGEKGEPGDDGpSGAEGPpGPQG	Collagen alpha- 1(II)	0.0100	−0.090
2280.97	26.16	ADGQpGAKGEQGEAGQKGDAGApGP	Collagen alpha- 1(II)	0.0116	−0.088
2412.11	27.18	RGGAGPPGpEGGKGAAGPpGpPGAAGTpG	Collagen alpha-1(III)	0.0377	0.072
1873.83	31.95	PPGpTGPGGDKGDTGPpGPQG	Collagen alpha-1(III)	0.0341	0.074
1141.51	26.28	EpGRDGVpGGpG	Collagen alpha-1(III)	0.0341	0.074
2130.97	32.98	GpTGpIGPpGpAGQPGDKGEGGAP	Collagen alpha-1(III)	0.0335	0.074
2507.13	22.82	ApGQNGEPGGkGERGAPGEkGEGGPpG	Collagen alpha-1(III)	0.0322	0.075
2663.21	23.57	NRGERGSEGSPGHpGQpGPPGpPGApGP	Collagen alpha-1(III)	0.0302	0.076
1794.80	24.01	GNDGApGKNGERGGpGGpGP	Collagen alpha-1(III)	0.0285	0.076
1531.68	39.25	GLpGPpGSNGNpGPpGP	Collagen alpha-1(III)	0.0271	0.077
1796.84	21.01	ApGPQGpRGDKGETGERG	Collagen alpha-1(III)	0.0243	0.079
883.41	23.48	PpGENGKpG	Collagen alpha-1(III)	0.0144	0.085
2264.05	22.67	KGDAGApGApGGKGDAGApGERGpPG	Collagen alpha-1(III)	0.0085	0.092
2135.96	25.80	GDAGApGApGGKGDAGApGERGPpG	Collagen alpha-1(III)	0.0080	0.092
1594.73	23.13	ApGGKGDAGApGERGpPG	Collagen alpha-1(III)	0.0029	0.104
2679.19	23.56	PGMPGADGpPGHPGKEGppGEKGGQGpPG	Collagen alpha-1(V)	0.0244	0.078
1522.73	22.99	KGDpGpAGLpGKDGpP	Collagen alpha-1(V)	0.0158	0.084
1176.56	26.86	KPGTDVFmGpP	Collagen alpha-1(XV)	0.0053	0.097
2226.96	33.46	GNSGEKGDQGFQGQPGFPGPpGP	Collagen alpha-1(XVI)	0.0046	0.099
3023.39	24.65	ppGAKGQEGAHGAPGAAGNPGAPGHVGAPGPSGpP	Collagen alpha-1(XXII)	0.0382	0.072
1540.74	39.81	GPpGVPGpPGpGGSPGLP	Collagen alpha-1(XXII)	0.0344	0.074
1536.72	19.91	KDGPnGPpGpPGTKGE	Collagen alpha-1(XXII)	0.0328	0.074
935.45	23.82	GRpGPpGPpG	Collagen alpha-1(XXVI)	0.0326	−0.075
1240.54	27.23	ApGEDGRpGPpGS	Collagen alpha-2(V)	0.0425	0.071
2480.21	23.24	EAGENQKQPEKNAGPTARTSATVP	C-X-C chemokine 16	0.0361	0.073
1728.76	36.62	ESVVLEPEAT	Fractalkine	0.0011	0.114
2272.24	23.91	SETAPAAPAAPAPAEKTPVKKKA	Histone H1.4	0.0070	0.094
937.46	34.16	PVQGQQQGP	Homeobox protein cut-like 1	0.0080	0.092
1276.71	19.96	KVVAGVANALAHK	Hemoglobin delta	0.0086	0.091
879.50	19.95	KLGHPDTL	Protein S100-A9	0.0088	0.091
2567.13	34.83	ATPLYINI	Protocadherin Fat 1	0.0131	0.086
2501.11	34.31	ASTAQASSSAASNNHQVGSGNDPWSA	Sorting nexin-9	0.0378	0.072
1294.62	19.43	ADHEGTHSTKRG	Fibrinogen alpha chain	0.0378	0.072
1013.37	25.06	cDDYRLcE	Matrix Gla Protein	0.0433	0.070
1159.61	26.41	SGSVIDQSRVL	Uromodulin	0.0440	−0.070
1099.49	28.06	DGGGSPKGDVDP	Sodium/potassium-transporting ATPase subunit gamma	0.0406	−0.071
1934.79	19.91	GSGGSSYGSGGGSYGSGGGGGGGRG	Keratin; type II cytoskeletal 1	0.0335	−0.074
1732.78	28.30	WVGTGASEAEKTGAQEL	Gelsolin	0.0126	−0.087
976.58	20.52	KELKFVTL	Prostatic acid phosphatase	0.0022	−0.107

**Table 5:** List of the 10 most highly ranked proteases that predicted with increased or decreased activity. Xcorr score is a correlation score which represents the predicted protease activity based on the frequency and urinary abundance of potential protease target peptides in PCa; # CS—Number of cleavage sites.

Protease	Uniprot ID	Symbol	# CS	Xcorr Score
Matrix metalloproteinase-20	O60882	MMP20	10	618.96
Matrix metalloproteinase-25	Q9NPA2	MMP25	73	283.10
Stromelysin-1	P08254	MMP3	93	260.70
Kallikrein-5	Q9Y337	KLK5	6	250.59
72 kDa type IV collagenase	P08253	MMP2	53	233.08
Calpain-2 catalytic subunit	P17655	CAPN2	146	231.49
Transmembrane protease serine 7	Q7RTY8	TMPRSS7	78	220.89
Caspase-1	P29466	CASP1	8	178.55
Macrophage metalloelastase	P39900	MMP12	134	162.13
Calpain-1 catalytic subunit	P07384	CAPN1	147	156.59
Cathepsin K	P43235	CTSK	77	−66.68
Meprin A subunit alpha	Q16819	MEP1A	109	−92.86
Kallikrein-4	Q9Y5K2	KLK4	18	−114.74
Prothrombin	P00734	F2	5	−192.73
Granzyme A	P12544	GZMA	13	−220.95
Plasminogen	P00747	PLG	13	−220.95
Cathepsin G	P08311	CTSG	36	−340.28
Serine protease hepsin	P05981	HPN	15	−363.28
Chymase	P23946	CMA1	5	−500.00
Tripeptidyl-peptidase 1	O14773	TPP1	5	−500.00

## Data Availability

Raw normalized proteomics data, along with the matched full clinical data applied in this manuscript and that are required to reproduce these analyses can be found at the Zenodo repository platform (DOI:10.5281/zenodo.7583241).

## Supplementary Materials

The following supporting information can be downloaded at: https://www.mdpi.com/article/10.3390/cancers15041166/s1. Supplementary Table S1 lists the full clinical and biochemical variables for the patients included in the development phase (n = 823). Supplementary Table S2 presents the list of the full clinical and biochemical variables for the patients included in the 2nd validation phase recruited at Innsbruck University Hospital. Supplementary Table S3 presents the output of the statistical comparison in the case-control study and the list of biomarkers that are integrated in the 181 Biomarker models. In addition, the list of twenty nine endogenous collagen fragments that were employed for normalization of the peptide intensities is also provided. Supplementary Table S4 presents the classification scores along with the details on the number of identified peptides and the mean intensity for the 50 CE-MS analysis of the standard urine sample as part of the reproducibility experiment. Supplementary Table S5 presents the output data as derived based on the correlation analysis in the development cohort of 823 patients. Supplementary Table S6 presents the estimation formulas for in silico prediction of the protease activity following the Proteasix analysis. Supplementary Figure S1 presents the distribution of the classification scores based on the 181 Biomarker model between the PCa patients (n = 138) and the non-PCa controls (n = 272) for the 1st validation set.
