# Supplementary figures and images for: Mass Spectrometry-Based Biomarkers to Detect Prostate Cancer: A Multicentric Study Based on Non-Invasive Urine Collection without Prior Digital Rectal Examination
Source: Cancers (Basel). 2023 Feb 11;15(4):1166. doi: 10.3390/cancers15041166 (PMC9954607; doi:10.3390/cancers15041166)

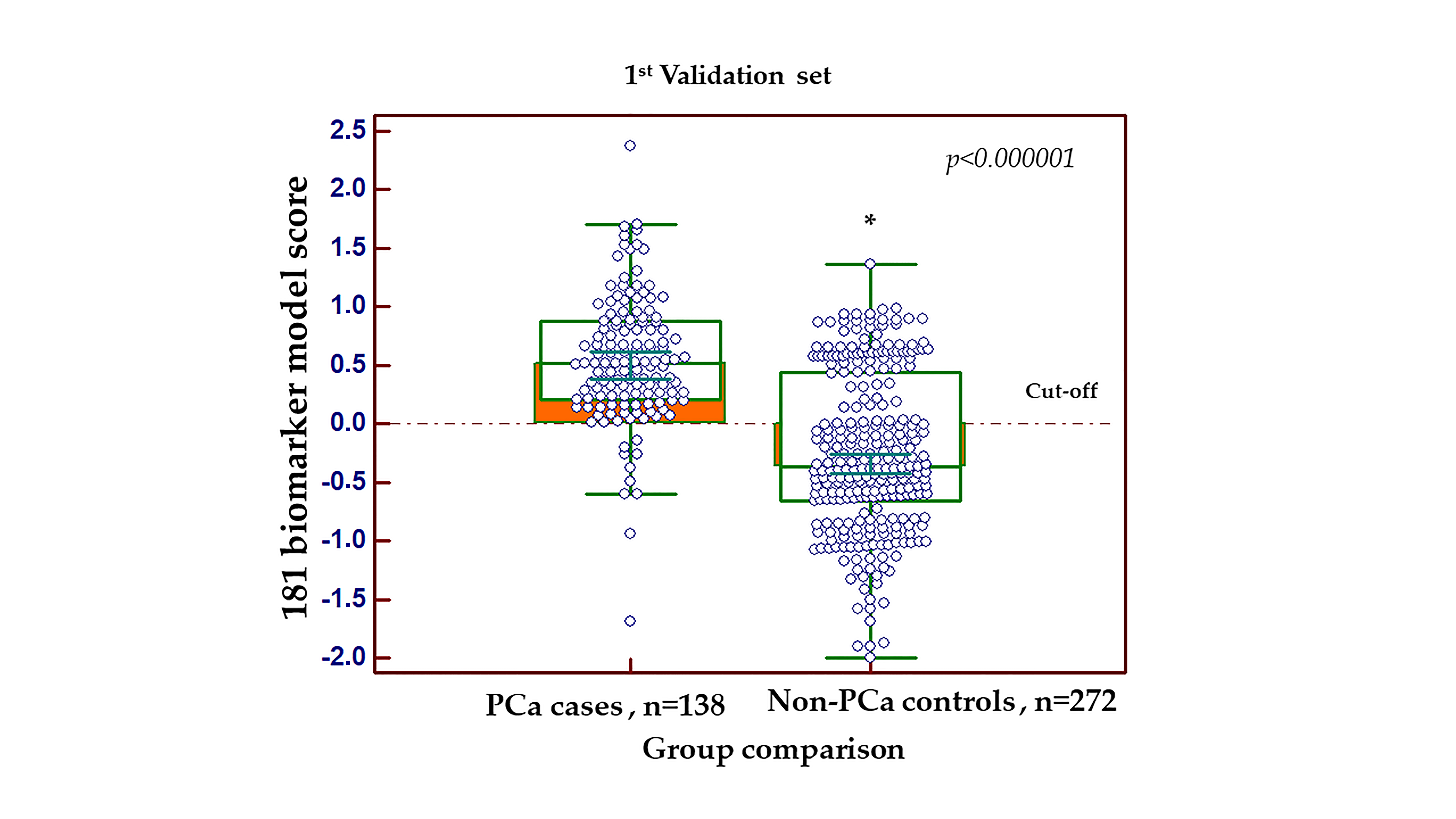

Supplement: Supplementary file 1 [file cancers-15-01166-s001.zip › Supplementary Figure S1.tif]
